# Salicin and Salicylic Acid in Rheumatism

**Published:** 1876-09

**Authors:** 


Salicin and Salicylic Acid in Rheumatism.—On their comparative merits, Dr. Maclagen, in the *British Medical Journal*, says:

"As I am probably the only person who has experience of both salicin and salicylic acid in the treatment of acute rheumatism, perhaps I may be allowed space for a few remarks on the merits of these two remedies.

"Which is the better remedy, salicin or salicylic acid? That each exercises a marvelous influence in cutting short an attack of acute rheumatism, there can be no doubt. I have used salicin or salicylic acid in every case of acute rheumatism which has come under my care since November, 1874, (a year and a half,) and invariably with the same result: a rapid cure of the disease. Seeing a patient suffering from acute rheumatism, I have no hesitation in assuring him that within forty-eight hours, possibly within twenty-four, he will be free from pain. This is a very different tale from any that can be told in connection with any other remedy.

"Salicin is the remedy which I used first, but I have not confined myself to it. When salicylic acid was first recommended as a febrifuge, I determined to give it a trial in acute rheumatism. In the first case in which I used it, ten grains were ordered every two hours. On seeing the patient after four doses had been taken, the general condition was a little better, but she complained much of the medicine 'burning her throat.' I urged her to continue it. This she did, and on the following morning the pain was less and the temperature had fallen from 102.3 to 101.1; but to the burning sensation in the throat was now added sickness. I omitted the salicylic acid, and gave the same dose of salicin, ten grains every two hours. The sickness ceased; the burning sensation in the throat disappeared, and by the following day the pain was entirely gone from the joints and the temperature had fallen to 98.8. She made a good recovery.

"This case well exemplifies what is the chief objection to salicylic acid—its tendency to produce irritation in the throat and stomach. I may have been unfortunate in my experience, but in every case in which I have given it this irritation has been complained of. All writers on the subject agree in referring to this irritation as one of its unpleasant effects. The salicylate of soda seems to give rise to the same disagreeable symptom. Salicin, on the other hand, never gives rise to any unpleasant effect. I have prescribed it the last year and a half in many different ailments, in doses ranging from five to thirty grains. I am probably within the mark when I say that I have thus given it to at least a hundred different people, and I cannot recall a single instance in which any disagreeable effect was produced.

"I have myself taken (by way of experiment) three doses of sixty grains—one in the forenoon, one in the afternoon, and one at night—without experiencing the least discomfort; but the smallest pinch of salicylic acid produces in me a feeling of heat and irritation in the throat, while a dose of ten grains gives rise to gastric irritation and a most unpleasant burning sensation in the fauces.

"Salicin is a pleasant bitter, and is best given mixed with a little water, flavored with syrup of orange if desired. In adequate doses, say fifteen grains every two hours, it cuts short an attack of rheumatic fever, without producing disagreeable effects. It should be continued in smaller doses during the first fortnight of convalescence.

"As remedial agents in acute rheumatism, salicin and salicylic acid seem to be equally efficacious; but the former has the advantage of producing no unpleasant effects. In time, too, it is sure to be much cheaper, a matter of some importance with a large class of sufferers from rheumatism."

